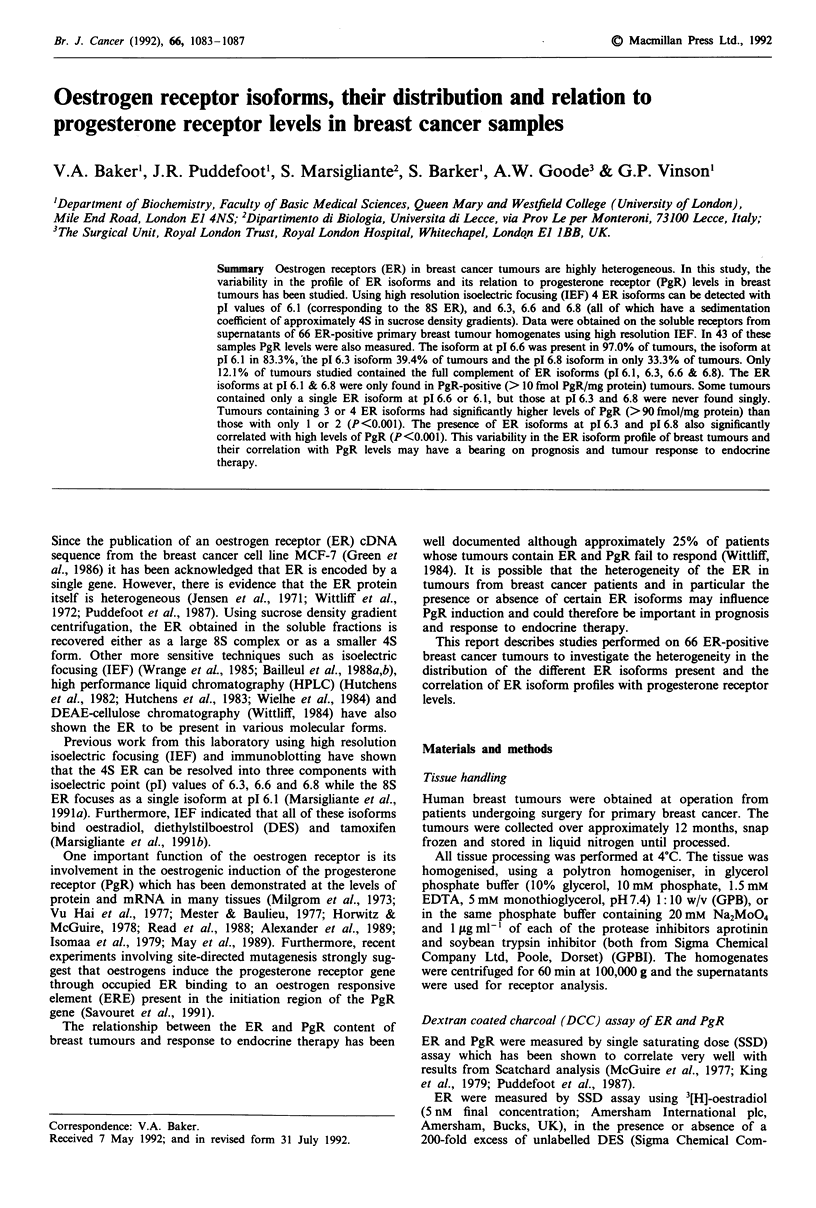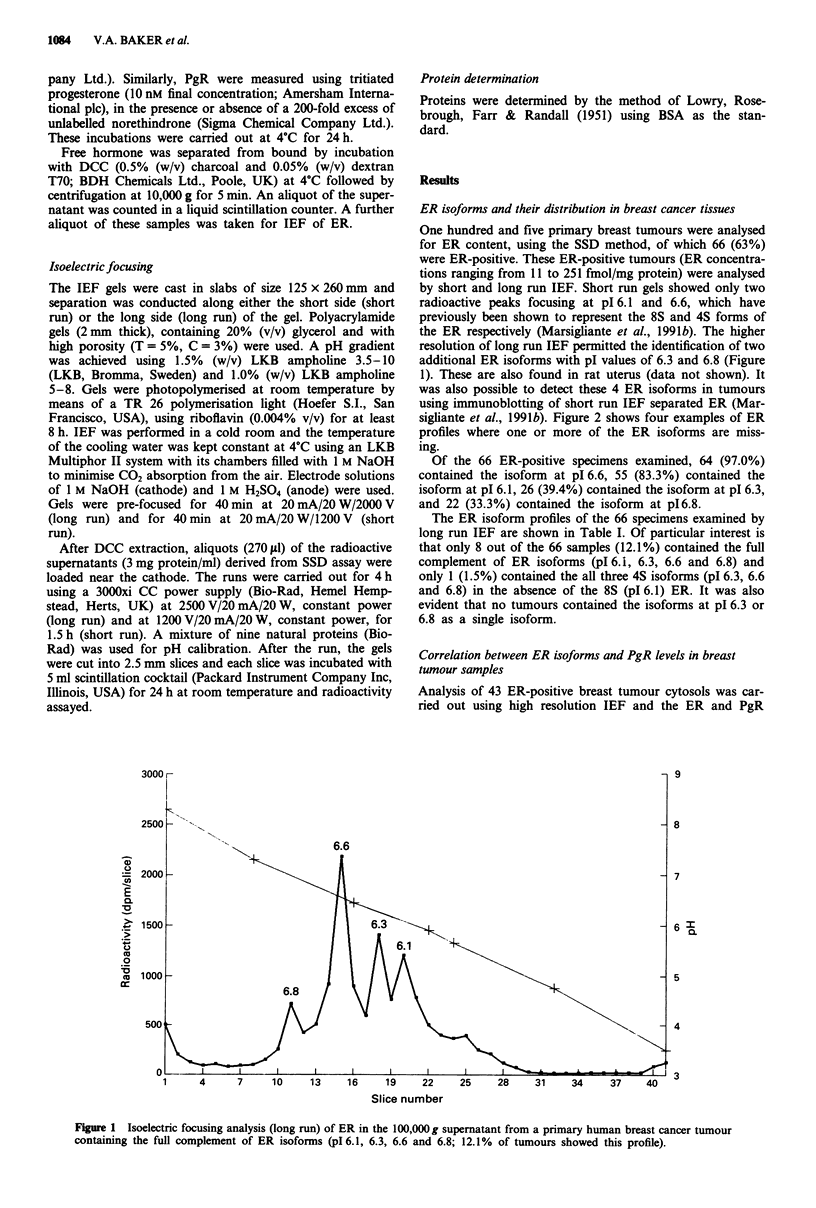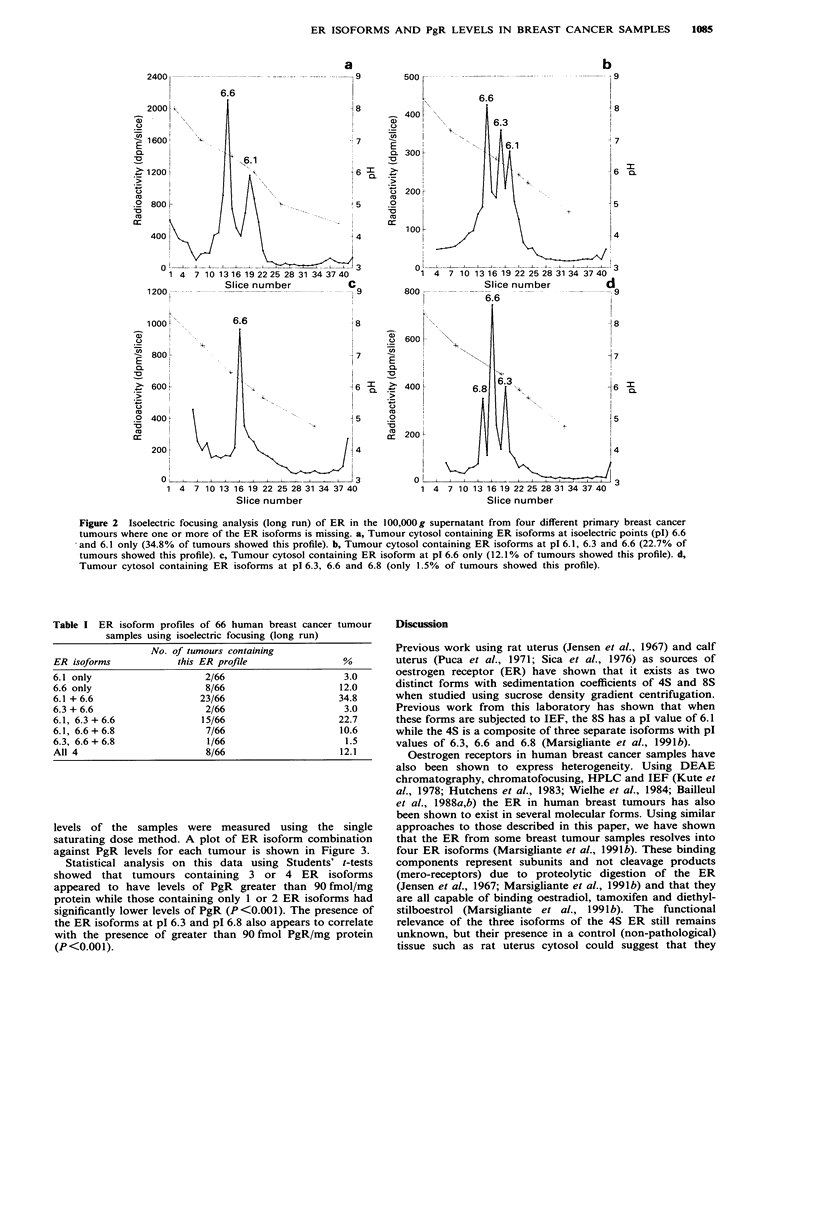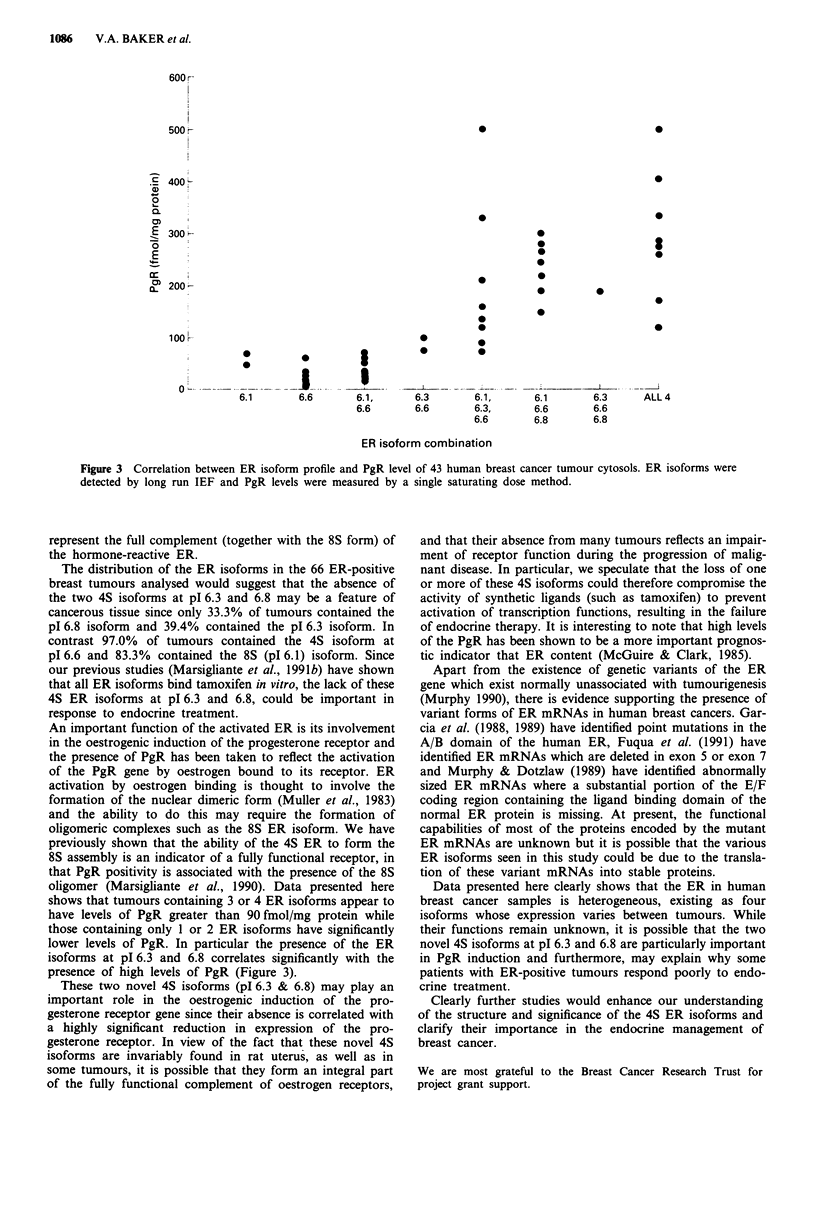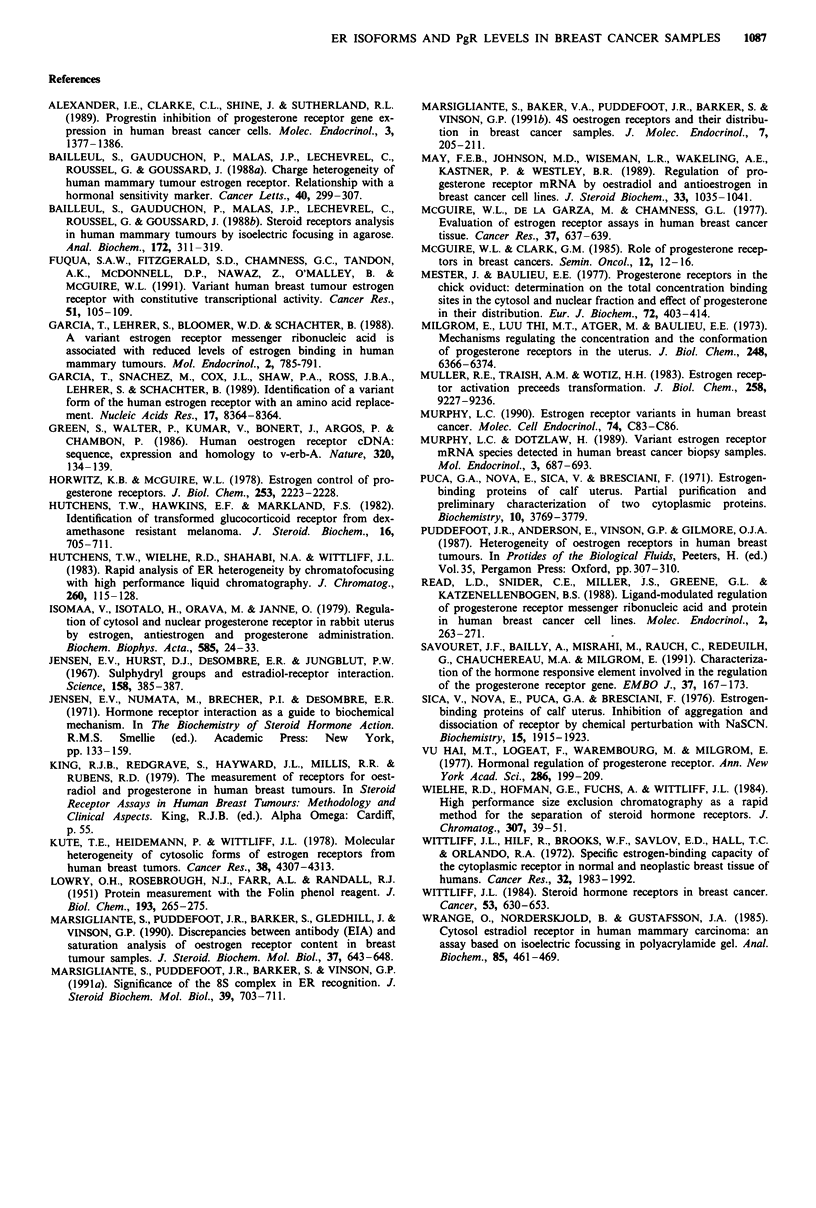# Oestrogen receptor isoforms, their distribution and relation to progesterone receptor levels in breast cancer samples.

**DOI:** 10.1038/bjc.1992.414

**Published:** 1992-12

**Authors:** V. A. Baker, J. R. Puddefoot, S. Marsigliante, S. Barker, A. W. Goode, G. P. Vinson

**Affiliations:** Department of Biochemistry, Faculty of Basic Medical Sciences, Queen Mary Westfield College, University of London, UK.

## Abstract

Oestrogen receptors (ER) in breast cancer tumours are highly heterogeneous. In this study, the variability in the profile of ER isoforms and its relation to progesterone receptor (PgR) levels in breast tumours has been studied. Using high resolution isoelectric focusing (IEF) 4 ER isoforms can be detected with pI values of 6.1 (corresponding to the 8S ER), and 6.3, 6.6 and 6.8 (all of which have a sedimentation pI values of 6.1 (corresponding to the 8S ER), and 6.3, 6.6 and 6.8 (all of which have a sedimentation coefficient of approximately 4S in sucrose density gradients). Data were obtained on the soluble receptors from supernatants of 66 ER-positive primary breast tumour homogenates using high resolution IEF. In 43 of these samples PgR levels were also measured. The isoform at pI 6.6 was present in 97.0% of tumours, the isoform at pI 6.1 in 83.3%, the pI 6.3 isoform 39.4% of tumours and the pI 6.8 isoform in only 33.3% of tumours. Only 12.1% of tumours studied contained the full complement of ER isoforms (pI 6.1, 6.3, 6.6 & 6.8). The ER isoforms at pI 6.1 & 6.8 were only found in PgR-positive (> 10 fmol PgR/mg protein) tumours. Some tumours contained only a single ER isoform at pI 6.6 or 6.1, but those at pI 6.3 and 6.8 were never found singly. Tumours containing 3 or 4 ER isoforms had significantly higher levels of PgR (> 90 fmol/mg protein) than those with only 1 or 2 (P < 0.001). The presence of ER isoforms at pI 6.3 and pI 6.8 also significantly correlated with high levels of PgR (P < 0.001). This variability in the ER isoform profile of breast tumours and their correlation with PgR levels may have a bearing on prognosis and tumour response to endocrine therapy.


Br. J. Cancer (1992), 66, 1083–1087 © Macmillan Press Ltd., 1992

### Oestrogen receptor isoforms, their distribution and relation to progesterone receptor levels in breast cancer samples

V.A. Baker[1], J.R. Puddefoot[1], S. Marsigliante[2], S. Barker[1], A.W. Goode[3] & G.P. Vinson[1]

[1]*Department of Biochemistry, Faculty of Basic Medical Sciences, Queen Mary and Westfield College (University of London), Mile End Road, London E1 4NS;* [2]*Dipartimento di Biologia, Universita di Lecce, via Prov Le per Monteroni, 73100 Lecce, Italy;* [3]*The Surgical Unit, Royal London Trust, Royal London Hospital, Whitechapel, London E1 1BB, UK.*

**Summary** Oestrogen receptors (ER) in breast cancer tumours are highly heterogeneous. In this study, the variability in the profile of ER isoforms and its relation to progesterone receptor (PgR) levels in breast tumours has been studied. Using high resolution isoelectric focusing (IEF) 4 ER isoforms can be detected with pI values of 6.1 (corresponding to the 8S ER), and 6.3, 6.6 and 6.8 (all of which have a sedimentation coefficient of approximately 4S in sucrose density gradients). Data were obtained on the soluble receptors from supernatants of 66 ER-positive primary breast tumour homogenates using high resolution IEF. In 43 of these samples PgR levels were also measured. The isoform at pI 6.6 was present in 97.0% of tumours, the isoform at pI 6.1 in 83.3%, the pI 6.3 isoform 39.4% of tumours and the pI 6.8 isoform in only 33.3% of tumours. Only 12.1% of tumours studied contained the full complement of ER isoforms (pI 6.1, 6.3, 6.6 & 6.8). The ER isoforms at pI 6.1 & 6.8 were only found in PgR-positive (> 10 fmol PgR/mg protein) tumours. Some tumours contained only a single ER isoform at pI 6.6 or 6.1, but those at pI 6.3 and 6.8 were never found singly. Tumours containing 3 or 4 ER isoforms had significantly higher levels of PgR (>90 fmol/mg protein) than those with only 1 or 2 ($P<0.001$). The presence of ER isoforms at pI 6.3 and pI 6.8 also significantly correlated with high levels of PgR ($P<0.001$). This variability in the ER isoform profile of breast tumours and their correlation with PgR levels may have a bearing on prognosis and tumour response to endocrine therapy.

Since the publication of an oestrogen receptor (ER) cDNA sequence from the breast cancer cell line MCF-7 (Green *et al.*, 1986) it has been acknowledged that ER is encoded by a single gene. However, there is evidence that the ER protein itself is heterogeneous (Jensen *et al.*, 1971; Wittliff *et al.*, 1972; Puddefoot *et al.*, 1987). Using sucrose density gradient centrifugation, the ER obtained in the soluble fractions is recovered either as a large 8S complex or as a smaller 4S form. Other more sensitive techniques such as isoelectric focusing (IEF) (Wrange *et al.*, 1985; Bailleul *et al.*, 1988*a,b*), high performance liquid chromatography (HPLC) (Hutchens *et al.*, 1982; Hutchens *et al.*, 1983; Wielhe *et al.*, 1984) and DEAE-cellulose chromatography (Wittliff, 1984) have also shown the ER to be present in various molecular forms.

Previous work from this laboratory using high resolution isoelectric focusing (IEF) and immunoblotting have shown that the 4S ER can be resolved into three components with isoelectric point (pI) values of 6.3, 6.6 and 6.8 while the 8S ER focuses as a single isoform at pI 6.1 (Marsigliante *et al.*, 1991*a*). Furthermore, IEF indicated that all of these isoforms bind oestradiol, diethylstilboestrol (DES) and tamoxifen (Marsigliante *et al.*, 1991*b*).

One important function of the oestrogen receptor is its involvement in the oestrogenic induction of the progesterone receptor (PgR) which has been demonstrated at the levels of protein and mRNA in many tissues (Milgrom *et al.*, 1973; Vu Hai *et al.*, 1977; Mester & Baulieu, 1977; Horwitz & McGuire, 1978; Read *et al.*, 1988; Alexander *et al.*, 1989; Isomaa *et al.*, 1979; May *et al.*, 1989). Furthermore, recent experiments involving site-directed mutagenesis strongly suggest that oestrogens induce the progesterone receptor gene through occupied ER binding to an oestrogen responsive element (ERE) present in the initiation region of the PgR gene (Savouret *et al.*, 1991).

The relationship between the ER and PgR content of breast tumours and response to endocrine therapy has been well documented although approximately 25% of patients whose tumours contain ER and PgR fail to respond (Wittliff, 1984). It is possible that the heterogeneity of the ER in tumours from breast cancer patients and in particular the presence or absence of certain ER isoforms may influence PgR induction and could therefore be important in prognosis and response to endocrine therapy.

This report describes studies performed on 66 ER-positive breast cancer tumours to investigate the heterogeneity in the distribution of the different ER isoforms present and the correlation of ER isoform profiles with progesterone receptor levels.

### Materials and methods

### *Tissue handling*

Human breast tumours were obtained at operation from patients undergoing surgery for primary breast cancer. The tumours were collected over approximately 12 months, snap frozen and stored in liquid nitrogen until processed.

All tissue processing was performed at 4°C. The tissue was homogenised, using a polytron homogeniser, in glycerol phosphate buffer (10% glycerol, 10 mM phosphate, 1.5 mM EDTA, 5 mM monothioglycerol, pH 7.4) 1:10 w/v (GPB), or in the same phosphate buffer containing 20 mM $Na_2MoO_4$ and 1 $\mu g\ ml^{-1}$ of each of the protease inhibitors aprotinin and soybean trypsin inhibitor (both from Sigma Chemical Company Ltd, Poole, Dorset) (GPBI). The homogenates were centrifuged for 60 min at 100,000 g and the supernatants were used for receptor analysis.

### *Dextran coated charcoal (DCC) assay of ER and PgR*

ER and PgR were measured by single saturating dose (SSD) assay which has been shown to correlate very well with results from Scatchard analysis (McGuire *et al.*, 1977; King *et al.*, 1979; Puddefoot *et al.*, 1987).

ER were measured by SSD assay using $^3$[H]-oestradiol (5 nM final concentration; Amersham International plc, Amersham, Bucks, UK), in the presence or absence of a 200-fold excess of unlabelled DES (Sigma Chemical Com-

Correspondence: V.A. Baker.
Received 7 May 1992; and in revised form 31 July 1992.

pany Ltd.). Similarly, PgR were measured using tritiated progesterone (10 nM final concentration; Amersham International plc), in the presence or absence of a 200-fold excess of unlabelled norethindrone (Sigma Chemical Company Ltd.). These incubations were carried out at 4°C for 24 h.

Free hormone was separated from bound by incubation with DCC (0.5% (w/v) charcoal and 0.05% (w/v) dextran T70; BDH Chemicals Ltd., Poole, UK) at 4°C followed by centrifugation at 10,000 g for 5 min. An aliquot of the supernatant was counted in a liquid scintillation counter. A further aliquot of these samples was taken for IEF of ER.

### *Isoelectric focusing*

The IEF gels were cast in slabs of size 125 × 260 mm and separation was conducted along either the short side (short run) or the long side (long run) of the gel. Polyacrylamide gels (2 mm thick), containing 20% (v/v) glycerol and with high porosity (T = 5%, C = 3%) were used. A pH gradient was achieved using 1.5% (w/v) LKB ampholine 3.5–10 (LKB, Bromma, Sweden) and 1.0% (w/v) LKB ampholine 5–8. Gels were photopolymerised at room temperature by means of a TR 26 polymerisation light (Hoefer S.I., San Francisco, USA), using riboflavin (0.004% v/v) for at least 8 h. IEF was performed in a cold room and the temperature of the cooling water was kept constant at 4°C using an LKB Multiphor II system with its chambers filled with 1 M NaOH to minimise $CO_2$ absorption from the air. Electrode solutions of 1 M NaOH (cathode) and 1 M $H_2SO_4$ (anode) were used. Gels were pre-focused for 40 min at 20 mA/20 W/2000 V (long run) and for 40 min at 20 mA/20 W/1200 V (short run).

After DCC extraction, aliquots (270 µl) of the radioactive supernatants (3 mg protein/ml) derived from SSD assay were loaded near the cathode. The runs were carried out for 4 h using a 3000xi CC power supply (Bio-Rad, Hemel Hempstead, Herts, UK) at 2500 V/20 mA/20 W, constant power (long run) and at 1200 V/20 mA/20 W, constant power, for 1.5 h (short run). A mixture of nine natural proteins (Bio-Rad) was used for pH calibration. After the run, the gels were cut into 2.5 mm slices and each slice was incubated with 5 ml scintillation cocktail (Packard Instrument Company Inc, Illinois, USA) for 24 h at room temperature and radioactivity assayed.

### *Protein determination*

Proteins were determined by the method of Lowry, Rosebrough, Farr & Randall (1951) using BSA as the standard.

### Results

### *ER isoforms and their distribution in breast cancer tissues*

One hundred and five primary breast tumours were analysed for ER content, using the SSD method, of which 66 (63%) were ER-positive. These ER-positive tumours (ER concentrations ranging from 11 to 251 fmol/mg protein) were analysed by short and long run IEF. Short run gels showed only two radioactive peaks focusing at pI 6.1 and 6.6, which have previously been shown to represent the 8S and 4S forms of the ER respectively (Marsigliante *et al.*, 1991*b*). The higher resolution of long run IEF permitted the identification of two additional ER isoforms with pI values of 6.3 and 6.8 (Figure 1). These are also found in rat uterus (data not shown). It was also possible to detect these 4 ER isoforms in tumours using immunoblotting of short run IEF separated ER (Marsigliante *et al.*, 1991*b*). Figure 2 shows four examples of ER profiles where one or more of the ER isoforms are missing.

Of the 66 ER-positive specimens examined, 64 (97.0%) contained the isoform at pI 6.6, 55 (83.3%) contained the isoform at pI 6.1, 26 (39.4%) contained the isoform at pI 6.3, and 22 (33.3%) contained the isoform at pI6.8.

The ER isoform profiles of the 66 specimens examined by long run IEF are shown in Table I. Of particular interest is that only 8 out of the 66 samples (12.1%) contained the full complement of ER isoforms (pI 6.1, 6.3, 6.6 and 6.8) and only 1 (1.5%) contained the all three 4S isoforms (pI 6.3, 6.6 and 6.8) in the absence of the 8S (pI 6.1) ER. It was also evident that no tumours contained the isoforms at pI 6.3 or 6.8 as a single isoform.

### *Correlation between ER isoforms and PgR levels in breast tumour samples*

Analysis of 43 ER-positive breast tumour cytosols was carried out using high resolution IEF and the ER and PgR

**Figure 1** Isoelectric focusing analysis (long run) of ER in the 100,000 *g* supernatant from a primary human breast cancer tumour containing the full complement of ER isoforms (pI 6.1, 6.3, 6.6 and 6.8; 12.1% of tumours showed this profile).

**Figure 2** Isoelectric focusing analysis (long run) of ER in the 100,000 *g* supernatant from four different primary breast cancer tumours where one or more of the ER isoforms is missing. **a**, Tumour cytosol containing ER isoforms at isoelectric points (pI) 6.6 and 6.1 only (34.8% of tumours showed this profile). **b**, Tumour cytosol containing ER isoforms at pI 6.1, 6.3 and 6.6 (22.7% of tumours showed this profile). **c**, Tumour cytosol containing ER isoform at pI 6.6 only (12.1% of tumours showed this profile). **d**, Tumour cytosol containing ER isoforms at pI 6.3, 6.6 and 6.8 (only 1.5% of tumours showed this profile).

**Table I** ER isoform profiles of 66 human breast cancer tumour samples using isoelectric focusing (long run)

| *ER isoforms* | *No. of tumours containing this ER profile* | *%* |
|---|---|---|
| 6.1 only | 2/66 | 3.0 |
| 6.6 only | 8/66 | 12.0 |
| 6.1 + 6.6 | 23/66 | 34.8 |
| 6.3 + 6.6 | 2/66 | 3.0 |
| 6.1, 6.3 + 6.6 | 15/66 | 22.7 |
| 6.1, 6.6 + 6.8 | 7/66 | 10.6 |
| 6.3, 6.6 + 6.8 | 1/66 | 1.5 |
| All 4 | 8/66 | 12.1 |

levels of the samples were measured using the single saturating dose method. A plot of ER isoform combination against PgR levels for each tumour is shown in Figure 3.

Statistical analysis on this data using Students' *t*-tests showed that tumours containing 3 or 4 ER isoforms appeared to have levels of PgR greater than 90 fmol/mg protein while those containing only 1 or 2 ER isoforms had significantly lower levels of PgR ($P<0.001$). The presence of the ER isoforms at pI 6.3 and pI 6.8 also appears to correlate with the presence of greater than 90 fmol PgR/mg protein ($P<0.001$).

### Discussion

Previous work using rat uterus (Jensen *et al.*, 1967) and calf uterus (Puca *et al.*, 1971; Sica *et al.*, 1976) as sources of oestrogen receptor (ER) have shown that it exists as two distinct forms with sedimentation coefficients of 4S and 8S when studied using sucrose density gradient centrifugation. Previous work from this laboratory has shown that when these forms are subjected to IEF, the 8S has a pI value of 6.1 while the 4S is a composite of three separate isoforms with pI values of 6.3, 6.6 and 6.8 (Marsigliante *et al.*, 1991*b*).

Oestrogen receptors in human breast cancer samples have also been shown to express heterogeneity. Using DEAE chromatography, chromatofocusing, HPLC and IEF (Kute *et al.*, 1978; Hutchens *et al.*, 1983; Wielhe *et al.*, 1984; Bailleul *et al.*, 1988*a*,*b*) the ER in human breast tumours has also been shown to exist in several molecular forms. Using similar approaches to those described in this paper, we have shown that the ER from some breast tumour samples resolves into four ER isoforms (Marsigliante *et al.*, 1991*b*). These binding components represent subunits and not cleavage products (mero-receptors) due to proteolytic digestion of the ER (Jensen *et al.*, 1967; Marsigliante *et al.*, 1991*b*) and that they are all capable of binding oestradiol, tamoxifen and diethylstilboestrol (Marsigliante *et al.*, 1991*b*). The functional relevance of the three isoforms of the 4S ER still remains unknown, but their presence in a control (non-pathological) tissue such as rat uterus cytosol could suggest that they

**Figure 3** Correlation between ER isoform profile and PgR level of 43 human breast cancer tumour cytosols. ER isoforms were detected by long run IEF and PgR levels were measured by a single saturating dose method.

represent the full complement (together with the 8S form) of the hormone-reactive ER.

The distribution of the ER isoforms in the 66 ER-positive breast tumours analysed would suggest that the absence of the two 4S isoforms at pI 6.3 and 6.8 may be a feature of cancerous tissue since only 33.3% of tumours contained the pI 6.8 isoform and 39.4% contained the pI 6.3 isoform. In contrast 97.0% of tumours contained the 4S isoform at pI 6.6 and 83.3% contained the 8S (pI 6.1) isoform. Since our previous studies (Marsigliante *et al.*, 1991*b*) have shown that all ER isoforms bind tamoxifen *in vitro*, the lack of these 4S ER isoforms at pI 6.3 and 6.8, could be important in response to endocrine treatment.

An important function of the activated ER is its involvement in the oestrogenic induction of the progesterone receptor and the presence of PgR has been taken to reflect the activation of the PgR gene by oestrogen bound to its receptor. ER activation by oestrogen binding is thought to involve the formation of the nuclear dimeric form (Muller *et al.*, 1983) and the ability to do this may require the formation of oligomeric complexes such as the 8S ER isoform. We have previously shown that the ability of the 4S ER to form the 8S assembly is an indicator of a fully functional receptor, in that PgR positivity is associated with the presence of the 8S oligomer (Marsigliante *et al.*, 1990). Data presented here shows that tumours containing 3 or 4 ER isoforms appear to have levels of PgR greater than 90 fmol/mg protein while those containing only 1 or 2 ER isoforms have significantly lower levels of PgR. In particular the presence of the ER isoforms at pI 6.3 and 6.8 correlates significantly with the presence of high levels of PgR (Figure 3).

These two novel 4S isoforms (pI 6.3 & 6.8) may play an important role in the oestrogenic induction of the progesterone receptor gene since their absence is correlated with a highly significant reduction in expression of the progesterone receptor. In view of the fact that these novel 4S isoforms are invariably found in rat uterus, as well as in some tumours, it is possible that they form an integral part of the fully functional complement of oestrogen receptors, and that their absence from many tumours reflects an impairment of receptor function during the progression of malignant disease. In particular, we speculate that the loss of one or more of these 4S isoforms could therefore compromise the activity of synthetic ligands (such as tamoxifen) to prevent activation of transcription functions, resulting in the failure of endocrine therapy. It is interesting to note that high levels of the PgR has been shown to be a more important prognostic indicator that ER content (McGuire & Clark, 1985).

Apart from the existence of genetic variants of the ER gene which exist normally unassociated with tumourigenesis (Murphy 1990), there is evidence supporting the presence of variant forms of ER mRNAs in human breast cancers. Garcia *et al.* (1988, 1989) have identified point mutations in the A/B domain of the human ER, Fuqua *et al.* (1991) have identified ER mRNAs which are deleted in exon 5 or exon 7 and Murphy & Dotzlaw (1989) have identified abnormally sized ER mRNAs where a substantial portion of the E/F coding region containing the ligand binding domain of the normal ER protein is missing. At present, the functional capabilities of most of the proteins encoded by the mutant ER mRNAs are unknown but it is possible that the various ER isoforms seen in this study could be due to the translation of these variant mRNAs into stable proteins.

Data presented here clearly shows that the ER in human breast cancer samples is heterogeneous, existing as four isoforms whose expression varies between tumours. While their functions remain unknown, it is possible that the two novel 4S isoforms at pI 6.3 and 6.8 are particularly important in PgR induction and furthermore, may explain why some patients with ER-positive tumours respond poorly to endocrine treatment.

Clearly further studies would enhance our understanding of the structure and significance of the 4S ER isoforms and clarify their importance in the endocrine management of breast cancer.

We are most grateful to the Breast Cancer Research Trust for project grant support.

### References

ALEXANDER, I.E., CLARKE, C.L., SHINE, J. & SUTHERLAND, R.L. (1989). Progrestin inhibition of progesterone receptor gene expression in human breast cancer cells. *Molec. Endocrinol.*, **3**, 1377–1386.

BAILLEUL, S., GAUDUCHON, P., MALAS, J.P., LECHEVREL, C., ROUSSEL, G. & GOUSSARD, J. (1988*a*). Charge heterogeneity of human mammary tumour estrogen receptor. Relationship with a hormonal sensitivity marker. *Cancer Letts.*, **40**, 299–307.

BAILLEUL, S., GAUDUCHON, P., MALAS, J.P., LECHEVREL, C., ROUSSEL, G. & GOUSSARD, J. (1988*b*). Steroid receptors analysis in human mammary tumours by isoelectric focusing in agarose. *Anal. Biochem.*, **172**, 311–319.

FUQUA, S.A.W., FITZGERALD, S.D., CHAMNESS, G.C., TANDON, A.K., MCDONNELL, D.P., NAWAZ, Z., O'MALLEY, B. & MCGUIRE, W.L. (1991). Variant human breast tumour estrogen receptor with constitutive transcriptional activity. *Cancer Res.*, **51**, 105–109.

GARCIA, T., LEHRER, S., BLOOMER, W.D. & SCHACHTER, B. (1988). A variant estrogen receptor messenger ribonucleic acid is associated with reduced levels of estrogen binding in human mammary tumours. *Mol. Endocrinol.*, **2**, 785-791.

GARCIA, T., SNACHEZ, M., COX, J.L., SHAW, P.A., ROSS, J.B.A., LEHRER, S. & SCHACHTER, B. (1989). Identification of a variant form of the human estrogen receptor with an amino acid replacement. *Nucleic Acids Res.*, **17**, 8364–8364.

GREEN, S., WALTER, P., KUMAR, V., BONERT, J., ARGOS, P. & CHAMBON, P. (1986). Human oestrogen receptor cDNA: sequence, expression and homology to v-erb-A. *Nature*, **320**, 134–139.

HORWITZ, K.B. & MCGUIRE, W.L. (1978). Estrogen control of progesterone receptors. *J. Biol. Chem.*, **253**, 2223–2228.

HUTCHENS, T.W., HAWKINS, E.F. & MARKLAND, F.S. (1982). Identification of transformed glucocorticoid receptor from dexamethasone resistant melanoma. *J. Steroid. Biochem.*, **16**, 705–711.

HUTCHENS, T.W., WIELHE, R.D., SHAHABI, N.A. & WITTLIFF, J.L. (1983). Rapid analysis of ER heterogeneity by chromatofocusing with high performance liquid chromatography. *J. Chromatog.*, **260**, 115–128.

ISOMAA, V., ISOTALO, H., ORAVA, M. & JANNE, O. (1979). Regulation of cytosol and nuclear progesterone receptor in rabbit uterus by estrogen, antiestrogen and progesterone administration. *Biochem. Biophys. Acta.*, **585**, 24–33.

JENSEN, E.V., HURST, D.J., DESOMBRE, E.R. & JUNGBLUT, P.W. (1967). Sulphydryl groups and estradiol-receptor interaction. *Science*, **158**, 385–387.

JENSEN, E.V., NUMATA, M., BRECHER, P.I. & DESOMBRE, E.R. (1971). Hormone receptor interaction as a guide to biochemical mechanism. In *The Biochemistry of Steroid Hormone Action*. R.M.S. Smellie (ed.). Academic Press: New York, pp. 133–159.

KING, R.J.B., REDGRAVE, S., HAYWARD, J.L., MILLIS, R.R. & RUBENS, R.D. (1979). The measurement of receptors for oestradiol and progesterone in human breast tumours. In *Steroid Receptor Assays in Human Breast Tumours: Methodology and Clinical Aspects*. King, R.J.B. (ed.). Alpha Omega: Cardiff, p. 55.

KUTE, T.E., HEIDEMANN, P. & WITTLIFF, J.L. (1978). Molecular heterogeneity of cytosolic forms of estrogen receptors from human breast tumors. *Cancer Res.*, **38**, 4307–4313.

LOWRY, O.H., ROSEBROUGH, N.J., FARR, A.L. & RANDALL, R.J. (1951) Protein measurement with the Folin phenol reagent. *J. Biol. Chem.*, **193**, 265–275.

MARSIGLIANTE, S., PUDDEFOOT, J.R., BARKER, S., GLEDHILL, J. & VINSON, G.P. (1990). Discrepancies between antibody (EIA) and saturation analysis of oestrogen receptor content in breast tumour samples. *J. Steroid. Biochem. Mol. Biol.*, **37**, 643–648.

MARSIGLIANTE, S., PUDDEFOOT, J.R., BARKER, S. & VINSON, G.P. (1991*a*). Significance of the 8S complex in ER recognition. *J. Steroid Biochem. Mol. Biol.*, **39**, 703–711.

MARSIGLIANTE, S., BAKER, V.A., PUDDEFOOT, J.R., BARKER, S. & VINSON, G.P. (1991*b*). 4S oestrogen receptors and their distribution in breast cancer samples. *J. Molec. Endocrinol.*, **7**, 205–211.

MAY, F.E.B., JOHNSON, M.D., WISEMAN, L.R., WAKELING, A.E., KASTNER, P. & WESTLEY, B.R. (1989). Regulation of progesterone receptor mRNA by oestradiol and antioestrogen in breast cancer cell lines. *J. Steroid Biochem.*, **33**, 1035–1041.

MCGUIRE, W.L., DE LA GARZA, M. & CHAMNESS, G.L. (1977). Evaluation of estrogen receptor assays in human breast cancer tissue. *Cancer Res.*, **37**, 637–639.

MCGUIRE, W.L. & CLARK, G.M. (1985). Role of progesterone receptors in breast cancers. *Semin. Oncol.*, **12**, 12–16.

MESTER, J. & BAULIEU, E.E. (1977). Progesterone receptors in the chick oviduct: determination on the total concentration binding sites in the cytosol and nuclear fraction and effect of progesterone in their distribution. *Eur. J. Biochem.*, **72**, 403–414.

MILGROM, E., LUU THI, M.T., ATGER, M. & BAULIEU, E.E. (1973). Mechanisms regulating the concentration and the conformation of progesterone receptors in the uterus. *J. Biol. Chem.*, **248**, 6366–6374.

MULLER, R.E., TRAISH, A.M. & WOTIZ, H.H. (1983). Estrogen receptor activation preceeds transformation. *J. Biol. Chem.*, **258**, 9227–9236.

MURPHY, L.C. (1990). Estrogen receptor variants in human breast cancer. *Molec. Cell Endocrinol.*, **74**, C83–C86.

MURPHY, L.C. & DOTZLAW, H. (1989). Variant estrogen receptor mRNA species detected in human breast cancer biopsy samples. *Mol. Endocrinol.*, **3**, 687–693.

PUCA, G.A., NOVA, E., SICA, V. & BRESCIANI, F. (1971). Estrogen-binding proteins of calf uterus. Partial purification and preliminary characterization of two cytoplasmic proteins. *Biochemistry*, **10**, 3769–3779.

PUDDEFOOT, J.R., ANDERSON, E., VINSON, G.P. & GILMORE, O.J.A. (1987). Heterogeneity of oestrogen receptors in human breast tumours. In *Protides of the Biological Fluids*, Peeters, H. (ed.) Vol. 35, Pergamon Press: Oxford, pp. 307–310.

READ, L.D., SNIDER, C.E., MILLER, J.S., GREENE, G.L. & KATZENELLENBOGEN, B.S. (1988). Ligand-modulated regulation of progesterone receptor messenger ribonucleic acid and protein in human breast cancer cell lines. *Molec. Endocrinol.*, **2**, 263–271.

SAVOURET, J.F., BAILLY, A., MISRAHI, M., RAUCH, C., REDEUILH, G., CHAUCHEREAU, M.A. & MILGROM, E. (1991). Characterization of the hormone responsive element involved in the regulation of the progesterone receptor gene. *EMBO J.*, **37**, 167–173.

SICA, V., NOVA, E., PUCA, G.A. & BRESCIANI, F. (1976). Estrogen-binding proteins of calf uterus. Inhibition of aggregation and dissociation of receptor by chemical perturbation with NaSCN. *Biochemistry*, **15**, 1915–1923.

VU HAI, M.T., LOGEAT, F., WAREMBOURG, M. & MILGROM, E. (1977). Hormonal regulation of progesterone receptor. *Ann. New York Acad. Sci.*, **286**, 199–209.

WIELHE, R.D., HOFMAN, G.E., FUCHS, A. & WITTLIFF, J.L. (1984). High performance size exclusion chromatography as a rapid method for the separation of steroid hormone receptors. *J. Chromatog.*, **307**, 39–51.

WITTLIFF, J.L., HILF, R., BROOKS, W.F., SAVLOV, E.D., HALL, T.C. & ORLANDO, R.A. (1972). Specific estrogen-binding capacity of the cytoplasmic receptor in normal and neoplastic breast tissue of humans. *Cancer Res.*, **32**, 1983–1992.

WITTLIFF, J.L. (1984). Steroid hormone receptors in breast cancer. *Cancer*, **53**, 630–653.

WRANGE, O., NORDERSKJOLD, B. & GUSTAFSSON, J.A. (1985). Cytosol estradiol receptor in human mammary carcinoma: an assay based on isoelectric focussing in polyacrylamide gel. *Anal. Biochem.*, **85**, 461–469.